# Supplementary material for: Novel Anti-Neuroinflammatory Properties of a Thiosemicarbazone–Pyridylhydrazone Copper(II) Complex
Source: Int J Mol Sci. 2022 Sep 14;23(18):10722. doi: 10.3390/ijms231810722 (PMC9505367; doi:10.3390/ijms231810722)
Supplement: Supplementary file 1 [file ijms-23-10722-s001.zip › ijms-1870147-supplementary.pdf]

1. Supplemental Figures

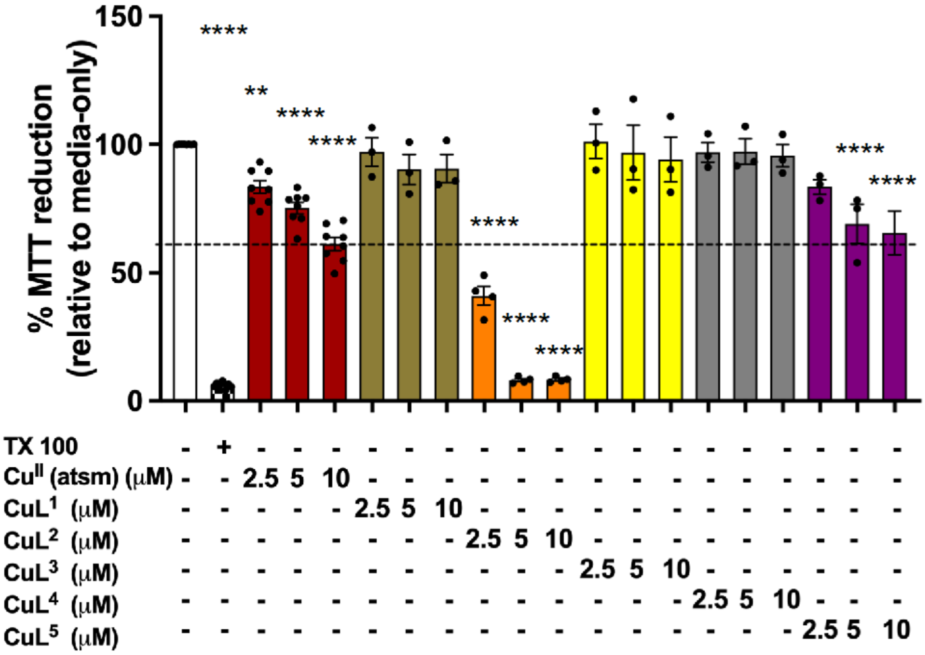

Figure S1. Dose-dependent toxicity assessments by MTT reduction assay exclude CuL^2 from further study. BE(2)-M17 cultures were incubated for 24h with metal-complexes (2.5-10μM). Cell viability was assessed by MTT reduction assay. Data are expressed as the percentage of MTT reduction relative to non-treated (media-only) controls and presented as mean ± S.E.M (n ≥ 3). Dotted line across graph represents the average % MTT reduction after treatment of BE(2)-M17 cultures with 10μM Cu^{II}(atsm) (~60% MTT reduction). Numbers on x-axis represent μM concentration of compound. TX 100= Triton X-100.

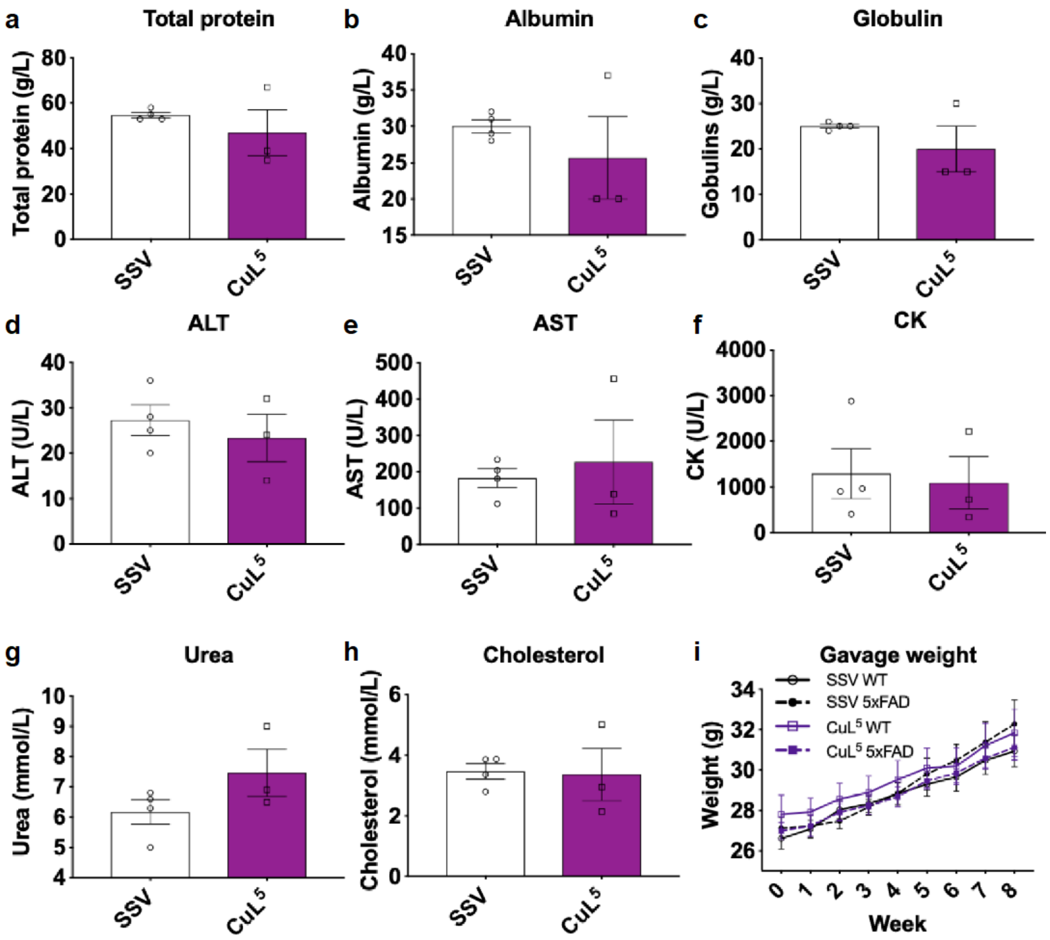

Figure S2. Treatment with CuL5 did not induce significant toxicity *in vivo*. For 1 week, non-transgenic WT mice were gavaged once daily with SSV or escalating doses (3-30mg/kg) of CuL5. At experimental end-point, blood samples were collected and analyses for the concentrations of a panel biomarkers, including (**a**) total protein, (**b**) albumin, (**c**) globulin, (**d**) ALT, (**e**) AST, (**f**) CK, (**g**) Urea and (**h**) cholesterol. These biomarkers were used as a proxy measure of the function and health of organs involved in drug metabolism, including the kidney and liver. The data for individual biomarkers are presented as the mean ± S.E.M from n=3-4 per treatment group and analysed for statistical significance using two-tailed Student's t-test. (**i**) SSV or CuL5 (30mg/kg) were delivered to WT or 5xFAD mice by oral gavage, once daily, for a period of 8 weeks. Individual mice were weighed once at the start of the trial (week 0) and also once a week throughout the treatment period. Across 9 time-points (weeks 0 - 8), the weights of animals in each treatment group are presented as mean ± S.E.M from n=5-8 per treatment group. The differences in the weights of the animals between treatment groups at each time-point were analysed for statistical significance using one-way ANOVA.

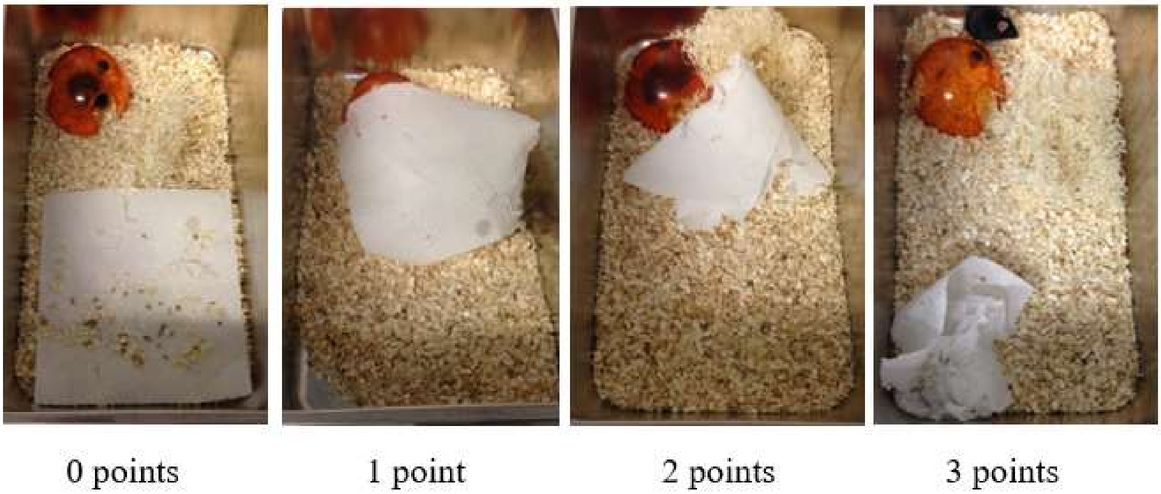

Figure S3. Images of exemplary nests and the nest-building rating scale used in the current study.

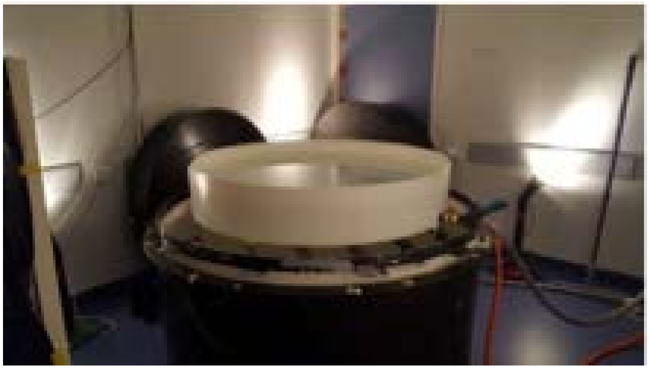

Figure S4. The pool and test conditions in Morris water maze (MWM).

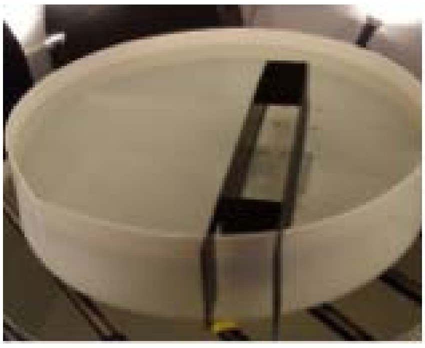

Figure S5. MWM practice trough.
